# Early childhood caries, obesity and anthropometric measurements: Is there a relationship?

**DOI:** 10.3389/fnut.2022.873562

**Published:** 2022-08-10

**Authors:** Karina Ferreira Rizzardi, Camila Lopes Crescente, Claudia Maria dos Santos Pereira Indiani, Carolina Steiner-Oliveira, Marinês Nobre-dos-Santos, Thaís Manzano Parisotto

**Affiliations:** ^1^Laboratory of Clinical and Molecular Microbiology, University São Francisco - USF, Bragança, Brazil; ^2^Pediatric Dentistry, Department of Pediatric Dentistry, Piracicaba Dental School, State University of Campinas - UNICAMP, Piracicaba, Brazil

**Keywords:** dental caries, children, anthropometry, obesity, preschool (kindergarten)

## Abstract

This brief research report explored the relationship among obesity, anthropometric measurements, and early childhood caries (ECC), in 3–5 years-old children. Three hundred and ninety-one Brazilian preschoolers were given anthropometric examinations for the assessment of general, peripheral, and central adiposity, by the following measures: body mass index (BMI), hip circumference, and waist circumference. Obesity status was determined by BMI according to WHO standards. Parent's age and BMI were assessed by questionnaire, and sucrose exposure was tracked by means of a food diary. For the assessment of ECC, dental examinations were performed according to modified WHO criteria. Also, the presence of dental biofilm in maxillary incisors was detected. A direct association between BMI and ECC was found in the bivariate analysis and the best possibility of logistic regression model showed that hip circumference (HC) values ≥62 centimeters (OR = 1.63*; p* = 0.033) jointly with the presence of dental biofilm (OR = 2.38*; p* = 0.000), children's ages ≥37 months (OR = 5.09*; p* = 0.012), and mothers younger than 35 years (OR = 1.96*; p* = 0.004) were significantly connected with ECC. In conclusion, peripheral adiposity (represented by HC) in young children was in fact associated with ECC. Thus, hip circumference might be a valuable tool for exploring the relationship between caries and obesity in the early years of life.

## Introduction

Controlling worldwide disorders like early childhood caries (ECC) and obesity remains one of the most important public health challenges (1, 2). Whereas obesity predisposes to cardiovascular problems, hypertension, dyslipidemia, and type-II diabetes (3), the consequences of caries in young children include pain, infection, chewing difficulty; and also poor oral-health-related quality-of-life conditions (2, 4).

The presence of active white-spot lesions or decayed/filled surfaces in children younger than 6 years old characterizes ECC, a chronic and multifactorial disease of rapid development and progression, affecting even the smooth surfaces of the mandibular incisors (5). With respect to obesity, a 0–5 years-old child with a body mass index (BMI) >99th percentile or > +3 height-for-weight score (Z-score) is classified as obese (6). Obesity is also a complex and chronic disorder (7, 8), manifested when more energy is consumed than is needed for the basal metabolism and physical activities.

Scientific evidence suggests ECC to be the most common oral disease in childhood, affecting more than 530 million children (9), with maternal circumstances, feeding practices, and oral-health- or infant-related oral health behaviors key risk indicators (10, 11). In the same way, childhood obesity is currently the most prevalent nutritional condition worldwide and is increasingly being cited as a health crisis reaching epidemic proportions (12). In 2020, in children under the age of 5, the number of obese children worldwide reached 39 million (13).

There has been growing interest in the relationship between dental caries and childhood obesity (14–17), disease sharing common risk indicators, such as feeding practices, for example. It is important to highlight that a common risk approach provides subsidies for more effective health promotion programs related to these disorders. In this respect, the early detection of ECC and obesity is essential for a better prognosis in future adolescence/adulthood, mainly because childhood is a key period for the establishment of healthy lifestyle patterns, and because oral health cannot be dissociated from general health (17). Thus, a study of shared indicators could lead to reduced levels of childhood obesity and dental caries in primary teeth. Thus, considering that the majority of studies in the scientific literature did not focus on the preschool-age period, we undertook the present study to explore the relationship between ECC and obesity in 3–5 years-old children.

## Materials and methods

### Ethical considerations

This observational study was approved by the Ethical Committee in Research of the São Francisco University (protocol number: CAAE-46107015.2.0000.5514) in full accordance with the World Medical Association Declaration of Helsinki. Parents or guardians of the children included in this study signed informed positive consent.

### Sample size

The sample size was calculated based on a previous study (14), considering the association between dental caries status and obesity (caries group: 18.1 ± 2.4; vs. caries-free group: 17.2 ± 1.8—weight/height ratio). The formula took into account an alpha value of 0.05 and a power of 98%, resulting in 358 children. Considering possible dropouts during the clinical procedures, the number was increased by 12.5% (*n* = 403).

### Sample characteristics

In total, between 2016 and 2017, informed positive consent was obtained for 403 children (3–5 years), good in general health, randomly selected from public preschools in Bragança Paulista-SP. Children who were absent from school when the dental examination/anthropometric measures were performed and who did not cooperate with the clinical exams were excluded. It is important to highlight that all children enrolled were of similar socio-economic status, and their parents had similar levels of education. The city of Bragança Paulista -SP has a human development index of 0.82 and a population of 170.533 inhabitants. The fluoride levels in the public water supply are 0.7 ppm.

### Presence of dental biofilm and clinical examination

Inspection for the presence or absence of dental biofilm was performed by visual examination of the buccal surfaces of maxillary incisors (18). After that, children had their teeth cleaned and dried with gauze, and the diagnosis of dental caries was performed under head-set light, with a clinical mirror and a ball-ended dental probe, according to the World Health Organization (19) criteria (decayed, missing, and filled surfaces of teeth—dmfs) modified by the inclusion of active white-spot lesions (18). Clinical examination was performed by two dentists, previously calibrated by a gold standard examiner, who received all the theoretical and practical instructions regarding the criteria to be used. During the calibration process, about 10% of the sample was re-examined with a 1-week interval. To guarantee a homogeneous interexaminer assessment, a Kappa statistic was calculated with a value of 0.86, indicating an excellent agreement between the examiners.

### Anthropometric measures

For anthropometric measurements, two examiners, previously calibrated, used a calibrated electronic scale (100 g of precision), a non-extensible measuring tape affixed to a wooden board at a 90° angle to the ground, and a movable piece as a headboard. For height measurements, the children were placed in an upright position, erect with the Camper Plane parallel to the floor and with their feet slightly apart. The preschoolers were weighed standing erect, on marked footprints in the center of the scale, and with arms stretched to the sides of the body. Even though children were weighed wearing only extremely light uniforms, without shoes, 100 g were subtracted from the total body mass. The body mass index (BMI = weight [kg]/height [m^2^]) was used to classify the children's nutritional status (eutrophic, thin, risk of overweight, overweight, and obese) according to the WHO guidelines. Children ages 5 years old or younger were considered obese when they were > +3 Z-score, overweight if ≥ +2 Z-score ≤ +3 Z-score, at risk of overweight if ≥ +1 Z-score ≤ +2 Z-score, eutrophic if ≥ −2 Z-score ≤ +1 Z-score, or underweight if < −2 Z-score (6).

In addition to weight and height measurements, waist circumference (WC), hip circumference (HC), and triceps skinfold thickness (TST) were taken by a calibrated and trained examiner based on standardized methods in anthropometry (20). Both HC and WC were measured by a stretch-resistant tape and were expressed in centimeters. In the interest of accuracy, children were required to stand relaxed, in a fasted state, with arms at the sides, feet together, and weight evenly distributed between them. For WC, the measurement was performed at the approximate midpoint between the lower margin of the last palpable rib and the top of the iliac crest, at the end of a normal expiration. With respect to the HC, the hip circumference measurement was taken around the widest portion of the buttocks. The TST was measured halfway between shoulder and elbow joints (midpoint of the posterior aspect of the humerus) on a child's right upper arm with the child standing upright. A digital scientific caliper (Prime Med DGi^®^- Brazil) with 0.1 mm of precision was used.

Waist circumference was used to estimate central adiposity. The TST and the HC were used to estimate peripheral adiposity.

### Evaluation of dietary sucrose exposure

Sugar intake was assessed by means of a food diary that parents and preschool health agents completed for three consecutive weekdays. Weekdays were chosen because on Saturdays or Sundays, the family might have more time to cook desserts or they may permit their children to eat more sweets, overestimating sugar consumption in the evaluation (18). The diary included the times of day when children ate and drank, as well as the contents of all meals/snacks. Moreover, if liquids containing sucrose were consumed in the baby bottle, this was also recorded. It should be reinforced that not only sucrose consumed at home was evaluated, but also that consumed in the preschools. This procedure increased the reliability of the collected data, and based on these, the daily frequency of sucrose exposure from food was manually calculated.

### Determination of the body mass index and age of parents

The age and BMI of parents were calculated based on the data obtained from the parents' responses to the following questions: “What is the age of the child's parents?” and “What are the height and weight of the child's mother and father?”.

### Statistical analysis

The results of ECC and obesity prevalence were expressed as percentages. In addition, the chi-square test was used to evaluate the possible association among ECC, obesity indicators, sucrose exposure, and social indicators. Then, the significant variables were selected to enter a stepwise forward regression model, to determine the best model possibility for ECC (dependent variable). In addition, obesity parameters were assessed one by one in the stepwise forward run to confirm the best model possibility and evidence of collinearity between them was examined. The independent variables were dichotomized based on their median/tercile values. The relationships between the dependent variable and the independent variables were expressed as odds ratio (OR). The model adjustment was checked by the Hosmer & Lemeshow test. The analyses were restricted to subjects with complete data and performed with the Statistical Package for the Social Sciences 17.0 (SPSS Inc., Chicago, IL, USA). The level of significance used was 5%.

## Results

A total number of 403 children were recruited, being five excluded due to non-collaboration with the clinical examinations and seven for being absent from school when the dental examination/anthropometric measures were performed. The final sample size comprised 391 children, of whom 53% were boys. The caries prevalence (dmfs > 0) in the studied population was 47%, and the mean value of decayed, missing, and filled surfaces was 3.03 (±6.23). The prevalence of obese individuals was 9%; however, when the overweight condition was considered, a percentage of 27% was achieved. Of the remaining children, 56% were eutrophic, 16% were at risk of being overweight, and 3% were underweight.

The association between ECC and clinical parameters for obesity and social indicators is shown in Tables 1, 2. The presence of dental biofilm, hip circumference, child's age, BMI of the child ≥19.96 (according to sex and age, all children enrolled in this study with BMI values higher than 19.96 were obese, as their Z-score were higher than +3), and age of the mother was significantly associated with ECC according to the chi-square test (*p* < 0.05; Table 1). In addition, Table 2 shows the best possibility of the stepwise forward logistic regression model, indicating that the main significant risk indicators associated with ECC in the present study were the presence of clinically visible biofilm in the maxillary incisors (OR = 2.38*; p* = 0.000), hip circumference ≥62 cm (OR = 1.63; *p* = 0.033), children 37 months of age or older (OR = 5.09*; p* = 0.012), and children whose mothers were younger than 35 years old (OR = 1.96*; p* = 0.004). Evidence of multicollinearity between adiposity parameters was checked during the choice of the best model possibility. It is important to highlight that the variance infraction factors (VIF) were smaller than 2, varying from 1.02 to 1.88, indicating no collinearity between them.

**Table 1:** Association between early childhood caries and clinical parameters for obesity, as well as social indicators.

		***Early Childhood Caries***	
		***Absent*** **(*n* %)**	***Present*** **(*n* %)**
Children's parameters	**Waist circumference (cm)**	*p* = 0.100	
	<53	124 (57)	95 (43)
	≥53	83 (48)	89 (52)
	**Hip circumference (cm)**	*p =* 0.006^*^	
	<62	147 (58)	106 (42)
	≥62	60 (43)	78 (57)
	**Triceps Skinfold (mm)**	*p =* 0.176	
	<10.95	167 (55)	138 (45)
	≥10.95	40 (47)	46 (53)
	**Body mass index-BMI (kg/m** ^**2**^ **)** ^**#**^	*p =* 0.028^*^	
	<19.96	191 (55)	157 (45)
	≥19.96	16 (37)	27 (63)
	**Age (months)**	*p =* 0.003^*^	
	<37	18 (86)	3 (14)
	≥37	189 (51)	181 (49)
	**Total Sucrose Exposure (times per day)**	*p =* 0.599	
	<6	160 (54)	138 (46)
	≥6	46 (51)	45 (49)
	**Baby bottle usage with sweetened liquids**	*p =* 0.596	
	Yes	70 (49)	82 (51)
	No	80 (50)	81 (50)
	**Dental Biofilm**	*p =* 0.000^*^	
	absent	82 (67)	40 (33)
	present	125 (46)	144 (54)
Parents' parameters	**Mother's BMI (kg/m** ^**2**^ **)**	*p =* 0.881	
	<30	147 (75)	50 (25)
	≥30	125 (75)	41 (25)
	**Mother's age (years)**	*p =* 0.001^*^	
	<35	122 (48)	134 (52)
	≥35	82 (65)	44 (35)
	**Father's BMI (kg/m** ^**2**^ **)**	*p =* 0.621	
	<30	137 (77)	40 (23)
	≥30	110 (80)	28 (20)
	**Father's age (years)**	*p =* 0.057	
	<35	94 (49)	97 (51)
	≥35	99 (59)	68 (41)

* Significant values derived from the Chi-square test (α = 0.05).

# BMI ≥ 19.96 kg/m^2^ in the studied preschoolers accounted for obesity. For the parameters: mother's age, mother's BMI, father's age, father's BMI, total sucrose exposure and baby bottle usage the non-response rate ranged from 1 to 20%.

**Table 2:** Multiple regression modeling of early childhood caries, based on dental biofilm presence, hip circumference, age and mother's ages.

		***Early Childhood caries***		***OR (95% CI)***	***p-value***
		***No (%)***	***Yes (%)***		
*Parameters*	**Hip circumference (cm)**				
	<62	147 (58)	106 (42)	1	0.033^*^
	≥62	60 (43)	78 (57)	1.63 (1.04–2.54)	
	**Dental Biofilm**				
	absent	82 (67)	40 (33)	1	0.000^*^
	present	125 (46)	144 (54)	2.38 (1.49–3.79)	
	**Age (months)**				
	<37	18 (86)	3 (14)	1	0.012^*^
	≥37	189 (51)	181 (49)	5.09 (1.44–17.97)	
	**Mother's age (years)**				
	<35	122 (48)	134 (52)	1.96 (1.24–3.09)	0.004^*^
	≥35	82 (65)	44 (35)	1	

* Significant values (α = 0.05). OR, odds ratio; CI, confidence interval. Model fitting information: −2 log likelihood, 40.50; chi-square, 37.98; degrees of freedom, 4; model's p-value, 0.000^*^;

Hosmer and Lemeshow test's p-value = 0.96.

## Discussion

The present study showed, for the first time in preschool children, to the best of our knowledge, that increased hip circumference (HC ≥ 62 cm) was associated with ECC (OR = 1.63; Table 2). Our results are particularly interesting because hip measurements could be performed to estimate peripheral adiposity, that is, excess levels of fat in the lower part of the human body, such as the leg, thigh, and gluteus maximus. The higher the HC, it might be suggested that the more fat could be accumulated in the peripheral region, and it was already demonstrated in children and adolescents that increased values of HC were found in the obese group in comparison to normal individuals (21). Intriguingly, it seems that adipokine dysregulation in gluteal fat (peripheral adiposity) contributes to both higher inflammation and insulin resistance (22), which are closely related to obesity. A recent study involving 6 year-old-children showed that the ones who had higher 'junk' food pattern scores were 0.44 cm higher in the (HC) (23). Also, another research investigating HC revealed that it was connected with higher sugar intake in 6–8 years old boys and with the percentage of total energy intake from sugar consumption in 6–8 years old girls (24).

In our investigation, the association was found between BMI and ECC in the Chi-square analysis (Table 1), corroborating results of previous studies (25, 26). Nevertheless, when the stepwise forward regression model was created, for joint evaluation of which significant studied indicators were most associated with dental caries, the best model possibility does not include the BMI, but the hip circumference (Table 2). Practical considerations might propose the use of hip circumference as an alternative to BMI, due to its simplicity and accuracy in obtention. Furthermore, since it is able to predict caries and is associated with obesity, the usefulness of hip circumference measurement as a first-step diagnostic tool could be a novelty justifying further investigation.

Key factors involving primary care and acting in both dental caries as well as obesity are sugar exposure and social environments. Lifestyle is influenced by high levels of free sugar intake, including mono- and disaccharides, mainly sucrose, added to foods by manufacturers, cooks, or consumers, as well as sugars naturally present in honey, syrups, fruit juices, and concentrated fruit juices (9, 27). Increased caloric intake (particularly fermentable carbohydrates/free sugars) may be associated with a higher prevalence of dental caries (28) and obesity in childhood (29), with great chances of this condition being perpetuated in adolescence and adulthood remaining a major burden in older ages.

The association between the frequency of sucrose exposure, baby bottle usage, and ECC could not be verified in the preschoolers of this study (Table 1). A possible explanation might be that these children have a similar socio-economic and cultural profile, besides attending a public preschool, where the majority of meals are provided. Another hypothesis would be that the mothers of caries-free children are theoretically more careful and thus may have completed the dietary chart more reliably than the mothers of children with caries lesions, who may have underestimated the consumption of sucrose-rich food at home as well as baby bottle usage (30), and this issue is not easy to resolve. In addition, a recent study (28) identified cariogenic beverages as significant risk factors for caries. Moreover, the study of Bernabé et al. (31), involving adults, has demonstrated that the amount of sugar intake could be more important than the frequency of consumption itself, but this was not evaluated in our study, being a possible limitation. Furthermore, a causal relationship could not be identified, due to the cross-sectional nature of the research design and possibly by the number of preschoolers included. Recent studies showing a significant connection between sucrose/sugar exposure and ECC encompassed a larger sample size, around 831–2181 children (32–34).

When the prevalence of ECC found in our study (47%) was compared with that of other countries, similar values could be observed in Africa (35) (44%) and in urban children of India (36) (47.2%). Regarding the prevalence of overweight together with obesity (27%), it was approximately 4.5 times higher than reported in the last Brazilian National Survey on Demographics and Health of Children and Women, performed more than 10 years before. This is a troubling finding, since excess weight may predict important pathologies such as obstructive sleep apnea, hypertension, type 2 diabetes-mellitus, dyslipidemia, and non-alcoholic fatty liver disease (29). Changes in lifestyle, such as increased consumption of foods with high energy density, sedentarism, and the evolution of the digital era, may partially explain the increased prevalence of obesity. The major problem is that the obese child has a considerable chance of remaining obese in adulthood (29).

We have observed that the presence of dental biofilm in the maxillary incisors continues to be a significant indicator of a child's being affected by dental caries lesions (OR = 2.38; Table 2). This evidence draws attention to and corroborates the findings of Parisotto et al. (37), who, in a follow-up study, considered the presence of dental biofilm as a risk factor for the development of dental caries, since dental biofilm harbors cariogenic bacteria and reflects poor oral hygiene. Additionally, a recent review pointed to dental plaque as a commonly identified predictor for ECC (38).

With respect to age, the younger the child, the lower the prevalence of ECC (OR = 5.09; Table 2). It is well-known that dental caries severity increases with age; the older the child, the longer the exposure of primary teeth to the prevailing cariogenic diet, as well as to pathological microorganisms and unsatisfactory oral hygiene, thus increasing the possibility of caries lesion development (37). Concerning the mother's age, it was observed that the younger the mother, the higher the caries index of the child (OR = 1.96; Table 2). This result suggests that younger mothers may have less experience since motherhood is a process of significant transformation in a woman's life.

Our results illustrate the dimensions of caries and obesity in early childhood, emphasizing the importance of the immediate implementation of effective health promotion programs targeting overweight and obese preschool children. Unfortunately, caries and obesity coexist, especially in children of low socioeconomic status.

It should be emphasized that preventing any kind of disease requires procedures of less complexity and lower cost compared with curative treatment. Nevertheless, since the oral cavity is part of the body, and since oral health cannot be dissociated from general health, a common preventive approach to risk indicators for ECC and obesity in young children seems more promising to prevent both diseases. Childhood is eminently suitable for prevention, and the early detection of risk indicators for these disorders is of prime importance.

Considering the limitations, one of them could be that only public preschools were included. If private schools had been included, differences in dietary habits and socio-economic backgrounds might have been more evident, and the association between frequency of sugar exposure and ECC might not have been impaired, even by possible untrustworthy reports from mothers or other caregivers. Moreover, longitudinal designs with powerful sample sizes should also be stimulated.

## Conclusion

In conclusion, the results of this study might suggest that peripheral adiposity in preschool children, represented by hip circumference, can be associated with ECC in the studied population, as well as with mother and child ages and the presence of dental biofilm. Thus, hip circumference may be a valuable tool in future investigations for exploration of the relationship between dental caries and obesity in the early years of life.

## Data availability statement

The raw data supporting the conclusions of this article will be made available by the authors, without undue reservation.

## Ethics statement

The studies involving human participants were reviewed and approved by University São Francisco. Written informed consent to participate in this study was provided by the participants' legal guardian/next of kin.

## Author contributions

CC and KR performed the collection of the clinical data, the data interpretation, and the writing of the manuscript. CI performed the collection of the clinical data and the writing of the first draft. MN-d-S and CS-O critically reviewed the manuscript, leading to the final version. TP was responsible for coordinating the study, for the design, the data interpretation, and also for writing the paper. All authors significantly contributed to the writing and approved the final version.

## Funding

This study was supported by FAPESP (2015/24600-2) and CNPq (409475/ 2016-5) grants.

## Conflict of interest

The authors declare that the research was conducted in the absence of any commercial or financial relationships that could be construed as a potential conflict of interest.

## Publisher's note

All claims expressed in this article are solely those of the authors and do not necessarily represent those of their affiliated organizations, or those of the publisher, the editors and the reviewers. Any product that may be evaluated in this article, or claim that may be made by its manufacturer, is not guaranteed or endorsed by the publisher.
